# Technical Factors Involved in the Measurement of Circulating MicroRNA Biomarkers for the Detection of Colorectal Neoplasia

**DOI:** 10.1371/journal.pone.0112481

**Published:** 2014-11-18

**Authors:** Atsushi Yamada, Mary A. Cox, Kristin A. Gaffney, Amber Moreland, C. Richard Boland, Ajay Goel

**Affiliations:** 1 Gastrointestinal Cancer Research Laboratory, Baylor Research Institute and Charles A. Sammons Cancer Center, Baylor University Medical Center, Dallas, TX, United States of America; 2 Department of Internal Medicine, Baylor University Medical Center, Dallas, TX, United States of America; University of Texas, MD Anderson Cancer Center, United States of America

## Abstract

**Background:**

Circulating miRNAs are emerging as promising blood-based biomarkers for colorectal and other human cancers; however, technical factors that confound the development of these assays remain poorly understood and present a clinical challenge. The aim of this study was to systematically evaluate the effects of factors that may interfere with the accurate measurement of circulating miRNAs for clinical purposes.

**Methods:**

Blood samples from 53 subjects, including routinely drawn serum samples, matched plasma from 30 subjects, and matched serum samples drawn before and after bowel preparation for colonoscopy from 29 subjects were collected. Additionally, 38 serum specimens stored in the clinical laboratory for seven days were used to test the stability of miRNAs. Hemolysis controls with serial dilutions of hemoglobin were prepared. RNA was extracted from serum, plasma or hemolyzed controls with spiked-in cel-miR-39, and levels of miR-21, miR-29a, miR-125b and miR-16 were examined by real-time RT-PCR. Hemolysis was measured by spectrophotometry.

**Results:**

The expression levels of miR-16 and the degree of hemolysis were significantly higher in plasma than in serum (P<0.0001). Measured miR-21, miR-29a, miR-125b and miR-16 expression increased with hemoglobin levels in hemolyzed controls. The degree of hemolysis in serum samples correlated significantly with the levels of miR-21 (P<0.0001), miR-29a (P = 0.0002), miR-125b (P<0.0001) and miR-16 (P<0.0001). All four miRNAs showed significantly lower levels in sera that had been stored at 4°C for seven days (P<0.0001). Levels of miR-21 (P<0.0001), miR-29a (P<0.0001) and miR-16 (P = 0.0003), and the degree of hemolysis (P = 0.0002) were significantly higher in sera drawn after vs. before bowel preparation.

**Conclusions:**

The measured levels of miRNAs in serum and plasma from same patients varied in the presence of hemolysis, and since hemolysis and other factors affected miRNA expression, it is important to consider these confounders while developing miRNA-based diagnostic assays.

## Introduction

MicroRNAs (miRNAs) are small non-coding RNA sequences of 19–25 nucleotides which predominantly act as translational repressors by interacting with 3′untranslated regions (UTR) of target mRNAs [1]. Physiologically, miRNAs play an important role in development, differentiation, apoptosis and proliferation; dysregulation of their expression has been implicated in human carcinogenesis [2], [3]. It has been reported that circulating cell-free miRNAs can be detected in serum and plasma in a highly stable form [4], [5], [6]. The stability of circulating miRNAs is attributed to their association with the Ago2 protein [7], [8], high-density lipoprotein (HDL) [9] or their presence within microparticles such as exosomes [10], [11], [12]; which collectively underscores their possible usefulness as blood-based biomarkers for human diseases. In fact, a number of miRNAs have been reported as blood-based biomarkers for diagnosis, determining prognosis, and predicting therapeutic responses in various types of cancer [1], [4], [5].

Despite accumulating evidence suggesting that circulating miRNAs are promising biomarkers, there are several technical issues that need to be addressed before these can be translated into clinical practice. In fact, there are confounding factors that can impact the measured levels of circulating miRNAs and potentially compromise their potential as disease biomarkers. [13] For example, there is no consensus on whether plasma or serum is a more reliable substrate for measuring circulating miRNAs. Mitchell et al. reported a strong correlation of miR-15b, miR-16, miR-19b and miR-24 levels between serum and plasma, and concluded that either serum or plasma would be suitable for investigation of miRNAs [4]. In contrast, another study showed higher levels of miR-15b, miR-16 and miR-24 in plasma than in serum. However, the expression levels of these miRNAs were reduced after a centrifugation step at 15,000 g, suggesting that higher concentrations of miRNAs in plasma were due to cellular contaminants such as platelets. [6] On the other hand, Wang et al. showed higher concentrations of miRNAs in serum than in plasma, and suggested the possible release of miRNAs into serum during coagulation [14]. While examining matched serum and plasma specimens from early stage non-small cell lung cancer (NSCLC) patients, investigators discovered 7 miRNAs with reduced expression in sera from NSCLC patients compared to healthy controls. However, no plasma miRNAs could accurately discriminate NSCLC patients from controls. Furthermore, a lack of correlation between the levels of miRNAs in serum and plasma was observed in this study. [15] As a result, there is no consensus regarding the superiority of using serum or plasma for investigating circulating miRNAs [13].

Hemolysis is another well-known confounding factor which can affect the circulating levels of various miRNAs. [6], [16], [17], [18], [19], [20], [21] Hemolysis involves the rupture of the red blood cells, and is recognized by pink discoloration of serum or plasma from the release of the red cell contents, including miRNAs, into the surrounding fluid. Since hemolysis commonly occurs during the process of preparing serum or plasma, it is important to consider its impact on measured levels of circulating biomarker miRNAs. Also, Baggish et al. reported that plasma levels of multiple miRNAs, including the biomarker miR-21, increased immediately after exhaustive exercise [22]. These results indicate that physical conditions at the time of blood drawing may also alter levels of circulating miRNAs. Moreover, while investigating novel diagnostic biomarkers for colorectal cancer, blood samples may be more readily accessible if drawn just before colonoscopy, i.e., after the bowel preparation for colonoscopy (colonoscopy prep), which involves gut lavage with a non-absorbable fluid. However, little is known about how colonoscopy preps affect on the levels of circulating miRNAs, or whether it is reasonable to use blood samples drawn via the intravenous catheter used during colonoscopy.

The aim of this study was to systematically address the missing gaps in knowledge and evaluate the effects of possible confounding factors in the measurement of circulating miRNAs. A better understanding of these technical issues is paramount for determining the usefulness of circulating miRNAs as blood-based biomarkers in clinical practice, and will help provide insights for a more robust standardization of the pre-analytical and analytical processes, and minimize the influence of these confounding factors for developing miRNA-based diagnostics for colorectal and other human cancers.

## Materials and Methods

### Study subjects

**Figure 1:**
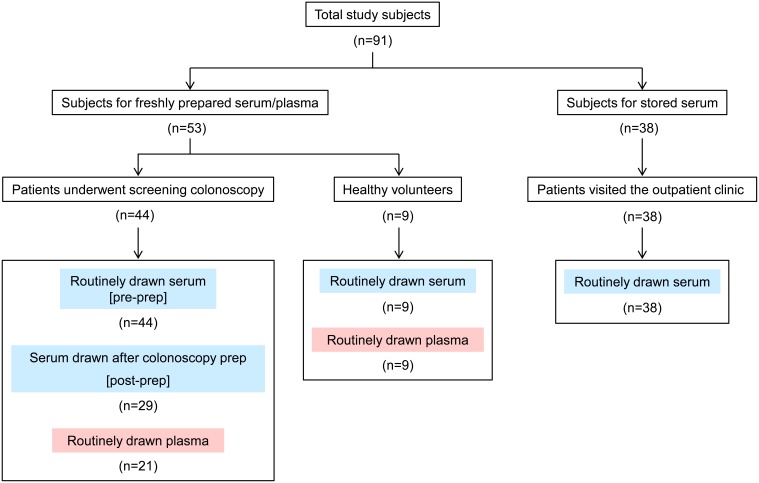
Study subjects and blood samples used in this study. The chart provides the description of patients and healthy volunteers from whom blood samples were collected under various conditions during the course of this study.

Blood samples were collected from a total of 91 subjects at the Baylor University Medical Center and its collaborating clinics. For 53 subjects, including 44 patients who underwent screening colonoscopy and 9 healthy volunteers, serum samples were prepared within a few hours after the blood drawing. Paired plasma samples were collected from 30 subjects among this group of patients and healthy subjects. For the 44 patients who underwent screening colonoscopy, blood samples were drawn prior to the colonoscopy prep, most often at the time of the initial consultation. To examine the effect of the prep on serum miRNA levels, serum samples were also drawn subsequent to the prep but prior to the colonoscopy in 29 patients. For these patients, the colonoscopy prep involved the oral ingestion of polyethylene glycol 3350 (NuLYTELY, Braintree Laboratories, Braintree, MA, USA). All serum and plasma samples from these patients and healthy subjects were immediately stored at –80°C until analysis. An additional 38 patients visited the outpatient clinic for various types of routine care, and serum samples were collected and stored at 4°C for seven days. These samples were collected without knowledge of the clinical background, and were used to investigate the stability of miRNA levels after storage. Samples used in this study are shown in **Figure 1**, and the characteristics of the study subjects are summarized in **Table S1**. All blood samples were drawn using butterfly needles, except for the post-colonoscopy prep samples, which were drawn from a peripheral intravenous catheter prior to initiation of any intravenous medications or fluids. BD Vacutainer SST Serum Separation Tubes for sera or K2 EDTA tubes for plasma (BD Biosciences, Franklin Lakes, NJ, USA) were used for blood collection. The study protocol was approved by the Institutional Review Board of the Baylor University Medical Center, Dallas and Kyoto University, Japan. Written informed consent was obtained from the colonoscopy patients and healthy volunteers, but the routine clinic patients’ samples were collected anonymously, and only linked to age and gender of the donor.

### Preparation of serially diluted hemolysed controls

Blood from a healthy volunteer was drawn into a K2 EDTA tube. A stock solution of hemolysis controls was made by adding 0.2 mL of whole blood from a K2 EDTA tube into 10 mL of distilled water to lyse the blood cells. PBS was added to the stock solution to generate serial dilutions of this sample.

### RNA extraction

RNA was extracted using miRNeasy Serum/Plasma Kit (QIAGEN, Hilden, Germany) according to the manufacturer’s instructions. Briefly, 250 µL of serum, plasma or hemolysis controls were thawed on ice and centrifuged at 16,000 g and at 4°C for 10 minutes to remove cellular debris. Thereafter 1000 µL of QIAzol Lysis Reagent was added to 200 µL of supernatant. After incubation for 5 minutes, 25 fmol of synthetic cel-miR-39 (Syn-cel-miR-39-3p miScript miRNA Mimic, QIAGEN) was spiked in. Total RNA including small RNA was extracted using QIAcube (QIAGEN) and eluted in 30 µL of RNase-free water. RNA was stored at −80°C until further usage.

### Real-time RT-PCR

Real-time RT-PCR was performed to examine the expression levels of miR-21, miR-29a, miR-125b and miR-16 using the TaqMan MicroRNA Assays (Applied Biosystems, Foster City, CA, USA) specific for each miRNA. MiR-21, miR-29a and miR-125b are known blood-based biomarkers for colorectal cancer [23], [24], [25], [26], and recently we also found these miRNAs as diagnostic biomarkers for colorectal neoplasms [manuscript in preparation]. MiR-16 has been used as an endogenous control [13], although it is also known to be abundantly present in red blood cells and its levels in serum and plasma can be elevated in hemolyzed samples [6], [16], [18], [19], [20], [21]. The cDNA was synthesized from a fixed volume (2 µL) of total RNA using the TaqMan MicroRNA Reverse Transcription Kit (Applied Biosystems) in a total volume of 15 µL under the following conditions: 16°C for 30 minutes, 42°C for 30 minutes, 85°C for 5 minutes and maintained at 4°C. Quantitative real-time PCR was conducted using the MicroRNA Assay Kit and the TaqMan Universal Master Mix II, no UNG (Applied Biosystems), and performed in duplicate on the StepOne Plus system (Applied Biosystems) under the following cycling conditions: 95°C for 10 minutes, followed by 40 cycles of 95°C for 15 seconds and 60°C for 1 minute. Cycle threshold (Ct) values were calculated using StepOne Software v2.3 (Applied Biosystems). Expression levels of miRNAs were normalized to those of cel-miR-39 and determined by the 2^−ΔCt^ method in which ΔCt was calculated as follows: ΔCt = Ct (miRNA of interest) – Ct (cel-miR-39).

### Assessment of the degree of hemolysis

Serum samples were visually inspected and the hemolysis score was determined as follows: 0, no sign of hemolysis; 1, slight hemolysis that cannot be ruled out due to dark yellow discoloring; 2, hemolysis is strongly suspected by orange to pink discoloring; 3, evident hemolysis with dark pink to red discoloring. Absorbance of serum samples was measured at 560 nm, 576 nm and 592 nm by spectrophotometry (Infinite M200 PRO, Tecan, Männedorf, Switzerland), and the degree of hemolysis was determined by the following formula: estimated hemoglobin level = 2*OD^576 nm^–OD^560 nm^–OD^592 nm^ [27].

### Statistical analysis

The Wilcoxon signed-rank test was used to compare the expression levels of miRNAs in paired serum and plasma samples, and paired serum samples drawn before and after colonoscopy prep. The differences in miRNA levels between non-paired samples were analyzed by the Mann-Whitney U test. Correlation between the degree of hemolysis and serial dilution of hemolyzed controls was assessed by Pearson’s correlation coefficient (R^2^), while correlation between the degree of hemolysis and serum miRNA levels was analyzed by Spearman’s rank correlation coefficient (ρ). All *P*-values were two-sided and a *P*-value <0.05 was considered significant. All analyses were carried out using JMP 10 (SAS institute Inc., Cary, NC).

## Results

### Comparison of miRNA expression levels in serum and plasma

**Figure 2:**
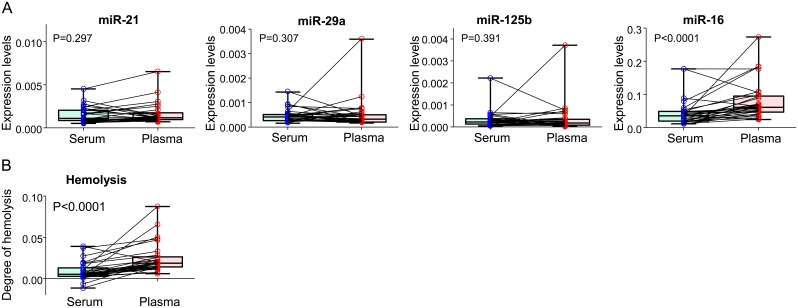
Measured miRNAs in matched serum and plasma. Expression levels of miR-21, miR-29a, miR-125b and miR-16 (**A**) and the degree of hemolysis (**B**) are illustrated. Absorbance of each serum sample at 560 nm, 576 nm and 592 nm was measured by spectrophotometry and the degree of hemolysis was calculated by the following formula: estimated hemoglobin level = 2*OD^576 nm^−OD^560 nm^−OD^592 nm^. Differences between serum and plasma were analyzed by the Wilcoxon signed-rank test.

First, we compared the expression levels of miRNAs in matched serum and plasma samples from 21 patients who underwent screening colonoscopy and 9 healthy volunteers. Levels of miR-21, miR-29a and miR-125b were not significantly different between matched serum and plasma, while miR-16 levels were significantly higher in plasma than in serum (**Figure 2A**). Because the levels of miR-16 are known to elevate by hemolysis, we compared the degree of hemolysis between serum and plasma. As shown in **Figure 2B**, the degree of hemolysis was significantly higher in plasma than in serum. Since the higher degree of hemolysis in our plasma samples appeared to be a confounder for measuring miRNA expression levels, we performed subsequent experiments using serum samples to address other technical issues.

### Effect of hemolysis on biomarker miRNAs

**Figure 3:**
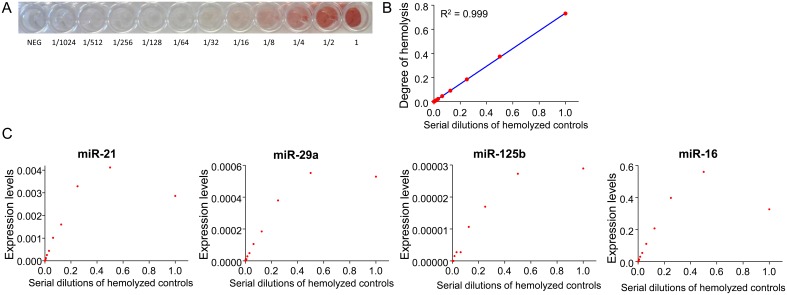
MiR-21, miR-29a, miR-125b and miR-16 in serial dilutions of hemolyzed control samples. **A:** Image of serial dilution of hemolyzed control samples. **B:** Correlation between dilutions of hemolyzed control samples and the degree of hemolysis is shown (Pearson’s correlation coefficient = R^2^). **C:** Levels of miR-21, miR-29a, miR-125b and miR-16 were elevated from baseline in hemolyzed control with 1/64^th^ dilution, and their levels increased along with the presumed hemoglobin concentration.

**Figure 4:**
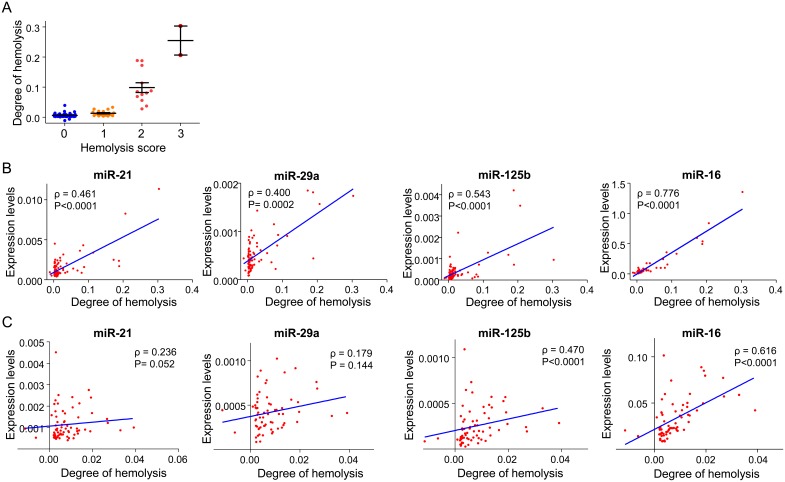
The degree of hemolysis in human serum samples. **A:** The relationship between the visual hemolysis score and the degree of hemolysis which was determined by spectrophotometry. The hemolysis score of each serum sample was visually designated as follows: 0, no sign of hemolysis; 1, slight hemolysis cannot be ruled out because of dark yellow discoloring; 2, hemolysis is strongly suspected by orange to pink discoloring; 3, evident hemolysis with dark pink to red discoloring. **B:** Correlation between the degree of hemolysis and serum levels of miR-21, miR-29a, miR-125b and miR-16. The Spearman’s rank correlation coefficient (ρ) is shown. **C:** Correlation between the degree of hemolysis and serum levels of miR-21, miR-29a, miR-125b and miR-16 in visually non-hemolysed sera (hemolysis score of 0 and 1). The Spearman’s rank correlation coefficient (ρ) is presented.

To explore the effect of hemolysis on miRNA biomarkers, we first prepared the serial dilution of hemolyzed controls made by adding PBS to the stock hemolysis solution (**Figure 3A**). As expected, the degree of hemolysis determined by spectrophotometry correlated well with serial dilution of controls (**Figure 3B**). Thereafter, we examined the expression levels of miR-21, miR-29a, miR-125b and miR-16 in hemolyzed controls. As shown in **Figure 3C**, all four miRNAs were detectable in hemolyzed controls, with a stepwise increase in their expression along with the presumed hemoglobin level. Levels of these miRNAs in hemolyzed controls showed clear elevation from the baseline at dilution of 1/64, in which discoloration was barely visible. Next, we evaluated the effect of hemolysis in the human serum samples. The degree of hemolysis determined by spectrophotometry was generally consistent with the hemolysis score designated by visual inspection (**Figure 4A**). Expression levels of all four examined miRNAs were significantly correlated with the degree of hemolysis in serum samples from 53 subjects (**Figure 4B**). Correlation between miR-125b and miR-16 expression and the degree of hemolysis were significant even while analyzing serum samples without any visible hemolysis (i.e. hemolysis score of 0 and 1, **Figure 4C**).

### Comparison of miRNA expression levels in freshly prepared and stored sera

**Figure 5:**
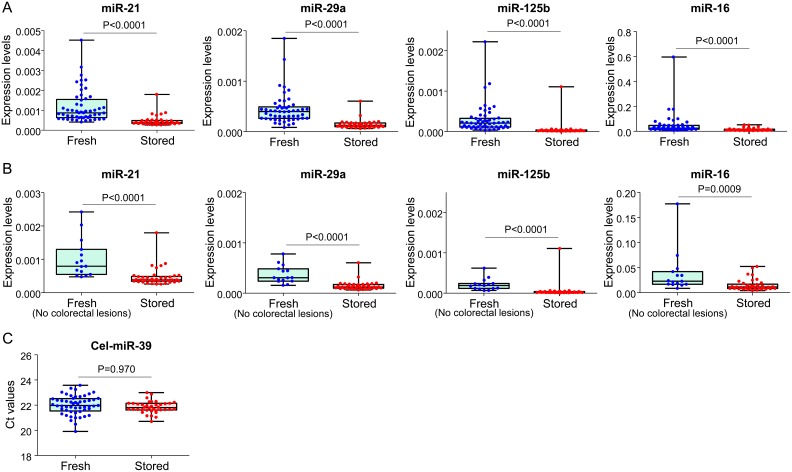
Comparison between freshly prepared and stored serum samples. **A–D:** Expression levels of miR-21 (**A**), miR-29a (**B**), miR-125b (**C**) and miR-16 (**D**) in freshly prepared and stored serum samples. **E–H:** Levels of four miRNAs were compared between freshly prepared sera from patients without known colorectal lesions and in stored sera. **I:** Ct values of spiked-in cel-miR-39 in freshly prepared and stored serum samples are shown. Differences were analyzed by the Mann-Whitney U test.

In clinical laboratories, blood samples are often stored for a period of time for re-examination and/or additional testing. Since these blood samples are discarded after a short period, 7 days in our collaborating pathology laboratory, these samples could be an important resource to researchers for developing novel blood-based biomarkers. To test the utility of these stored samples for investigating miRNA biomarkers, we examined the miRNA expression levels in stored and freshly prepared sera. As shown in **Figure 5A**, expression levels of all four miRNAs were remarkably lower in serum samples that had been stored at 4°C for 7 days within serum collection tubes. These differences persisted even when we compared stored vs. freshly prepared serum from the screening colonoscopy subjects who had no colorectal lesions (**Figure 5B**). Since the Ct values for spiked-in cel-miR-39 did not differ significantly (**Figure 5C**), the differences in the expression of endogenous miRNAs are likely attributable to steps that occur prior to RNA extraction, including storage for 7 days at 4°C in blood collection tubes.

### Effect of colonoscopy prep on serum miRNA expression levels

**Figure 6:**
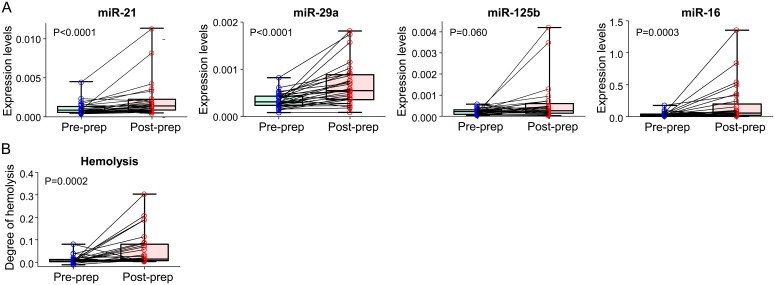
Comparison between serum samples drawn before and after a colonoscopy prep. Expression levels of miR-21, miR-29a, miR-125b and miR-16 (**A**) and the degree of hemolysis (**B**) are shown. Differences between serum samples drawn before (pre-prep) and after (post-prep) colonoscopy prep were analyzed by the Wilcoxon signed-rank test.

To investigate whether the colonoscopy prep affects the expression of circulating miRNAs, we evaluated serum levels of miR-21, miR-29a, miR-125b and miR-16 in matched samples drawn before and after the colonoscopy prep. As shown in **Figure 6A**, levels of miR-21, miR-29a and miR-16 were significantly higher in sera drawn after the prep. This might at least partly attribute to hemolysis since the degree of hemolysis was significantly higher in the post-prep sera (**Figure 6B**).

## Discussion

In recent years, abundant evidence has accumulated highlighting the promising potential of circulating miRNAs as blood-based biomarkers for human cancers. However, since various technical factors can potentially affect the levels of circulating miRNAs, the impact of confounders on the reliable measurement of miRNA biomarkers must be elucidated prior to their adoption for clinical use. In this study, we systematically evaluated the effect of factors that alter the levels of circulating miRNA biomarkers in the context of colorectal neoplasia.

First, we compared miRNA expression levels in matched serum and plasma collected from the same subjects at the same time. Among the four miRNAs examined, the expression levels of miR-16 were significantly higher in plasma than in serum. This difference was evident even during the pre-analytical steps including high-speed centrifugation to remove cellular debris in an attempt to minimize differences in miRNA levels between serum and plasma samples as suggested in previous reports [6]. During our analysis of a limited number of miRNAs, we identified differences only in miR-16 levels; nonetheless, our data raises the possibility that miRNA expression in plasma specimens is generally higher even when one takes precautions during plasma preparation. These findings highlight that data on miRNA biomarkers should be carefully interpreted based upon whether serum or plasma was used for analysis. This is particularly important when using miR-16 as an internal control.

Hemolysis is a well-known confounding factor for circulating miRNA measurement. Here, we illustrate that elevated miR-21, miR-29a, miR-125b and miR-16 expression was easily detected in hemolyzed controls. Since we used PBS to dilute our stock solution, and the levels of four miRNAs were elevated as a function of the hemoglobin level, it is likely that these miRNAs originated from the lysed red blood cells. This is consistent with previous reports [6], [16], [17], [18], [19], [20], [21], and suggests that circulating miRNA biomarker results might be erroneously interpreted in hemolyzed serum or plasma samples. In fact, expression levels of miR-21, miR-29a, miR-125b and miR-16 showed a significant correlation with the degree of hemolysis in serum samples from subjects without apparent colorectal lesions.

A simple compensating strategy could be to exclude any samples with visually recognizable hemolysis from the final analysis. However, this approach might raise other critical issues. First, among 53 routinely drawn serum samples by standard phlebotomy using a butterfly needle, 4 samples (7.5%) had visually recognizable hemolysis (i.e. a hemolysis score of 2 or 3). If we exclude all visibly hemolyzed samples, we could miss a significant number of patients who suffer from the disease of interest. Second, hemolysis can affect the levels of miRNA biomarkers even when the degree of hemolysis is subtle, and may not be visually apparent. In fact, serum levels of miR-125b and miR-16 significantly correlated with the degree of hemolysis even in samples with no visually recognizable hemolysis (i.e. a visual hemolysis score of 0 and 1). Thus, it might be better to evaluate the degree of hemolysis by sensitive objective methods such as spectrophotometry, rather than simple visual inspection. One possible solution to overcome the interference by hemolysis may be to correct the levels of miRNA biomarkers based on the degree of hemolysis. Further studies are required to substantiate these possibilities.

We also found that the serum samples stored for 7 days in our clinical laboratory had remarkably lower levels of all four tested miRNAs compared with freshly prepared sera. Many of the latter serum specimens came from patients with colorectal polyps. Although levels of miRNA biomarkers may be increased in patients with colorectal polyps, these differences were still obvious when we compared stored sera vs. fresh sera from patients who had no known colorectal lesions. These results indicate that although miRNAs are considered to be relatively stable in bodily fluids including serum and plasma [4], [5], storage at 4°C for 7 days seemed to result in miRNA degradation, underlining the caution one must exercise during analysis of circulating miRNA biomarkers. Prolonged storage time without freezing, with cellular components and/or in serum collection tubes, may contribute to the degradation of miRNAs. Therefore, immediate separation of serum and storage without cellular component at −80°C may be required to investigate biomarker miRNAs, as recommended recently [13].

To address the effect of colonoscopy prep on miRNA biomarkers, we utilized matched serum samples drawn before and after the colonoscopy prep. Interestingly, we showed a significant increase in the levels of miR-21, miR-29a and miR-16 in post-prep than in pre-prep sera. There are several possible explanations for this finding. First, the degree of hemolysis was significantly higher in post-prep samples, and the hemolysis may have resulted in elevated levels of serum miRNAs. Different phlebotomy techniques may have influenced the degree of hemolysis, since blood samples after the colonoscopy prep were drawn from a peripheral intravenous catheter rather than through butterfly needles, which were used for most of other blood samples. Second, frequent defecation during the prep may alter body homeostasis, which can also affect levels of circulating miRNAs. Also, insufficient water intake during the prep can cause dehydration and influence the measured miRNA levels. Since we used same amount of starting serum volume of RNA extracted from each subject, as well as used spiked-in cel-miR-39 as a normalizer for real-time RT-PCR measurements, dehydration may significantly influence the levels of circulating miRNAs. Finally, the stress of the diagnostic procedure about to be performed may have influenced miRNA levels – although we have no evidence that this phenomenon occurs.

In conclusion, in this study we provide a systematic evaluation of several critical factors that can have a significant impact on the measurement of circulating miRNA biomarkers. Our data clearly highlight that since hemolysis occurs frequently during blood collection, and that such samples are not always stored under uniform conditions, a better understanding of these confounders should be extremely important for the interpretation of clinically meaningful results that utilize circulating miRNA biomarkers. Our findings underscore the need for developing approaches, technical or even statistical, to account for the degree of hemolysis and its effect on circulating biomarker miRNAs. In addition, blood samples should be drawn under standardized conditions and techniques that minimize the influence of such confounders while developing miRNA biomarkers for colorectal and other human malignancies.

## Supporting Information

Table S1 Characteristics of study subjects.
(XLSX)
